# Influence of aging and gadolinium exposure on T1, T2, and T2*-relaxation in healthy women with an increased risk of breast cancer with and without prior exposure to gadoterate meglumine at 3.0-T brain MR imaging

**DOI:** 10.1007/s00330-021-08069-4

**Published:** 2021-07-03

**Authors:** Kathrin Barbara Krug, Christina Jane Burke, Kilian Weiss, Pascal A. T. Baltzer, Kerstin Rhiem, David Maintz, Marc Schlamann, Martin Hellmich

**Affiliations:** 1 Department of Diagnostic and Interventional Radiology, University Hospital of Cologne, Kerpener Straße 62, 50937 Cologne, Germany; 2 Philips Medical Systems, Hamburg, Germany; 3 Department of Biomedical Imaging and Image-guided Therapy, Division of General Radiology, Allgemeines Krankenhaus, Medical University of Vienna, Vienna, Austria; 4 Center for Hereditary Breast and Ovarian Cancer, Center for Integrated Oncology (CIO), Faculty of Medicine and University Hospital of Cologne, University of Cologne, Cologne, Germany; 5 Institute of Medical Statistics and Bioinformatics, University of Cologne, Cologne, Germany

**Keywords:** Gadolinium, Contrast media, Middle aged, Globus pallidus, Cerebellar nuclei

## Abstract

**Objectives:**

We examined the effects of aging and of gadolinium-based contrast agent (GBCA) exposure on MRI measurements in brain nuclei of healthy women.

**Methods:**

This prospective, IRB-approved single-center case-control study enrolled 100 healthy participants of our high-risk screening center for hereditary breast cancer, who had received at least six doses of macrocyclic GBCA (exposed group) or were newly entering the program (GBCA-naïve group). The cutoff “at least six doses” was chosen to be able to include a sufficient number of highly exposed participants. All participants underwent unenhanced 3.0-T brain MRI including quantitative T1, T2, and R2* mapping and T1- and T2-weighted imaging. The relaxation times/signal intensities were derived from region of interest measurements in the brain nuclei performed by a radiologist and a neuroradiologist, both board certified. Statistical analysis was based on descriptive evaluations and uni-/multivariable analyses.

**Results:**

The participants (exposed group: 49, control group: 51) were aged 42 ± 9 years. In a multivariable model, age had a clear impact on R2* (*p* < 0.001–0.012), T2 (*p* = 0.003–0.048), and T1 relaxation times/signal intensities (*p* < 0.004–0.046) for the majority of deep brain nuclei, mostly affecting the substantia nigra, globus pallidus (GP), nucleus ruber, thalamus, and dentate nucleus (DN). The effect of prior GBCA administration on T1 relaxation times was statistically significant for the DN, GP, and pons (*p* = 0.019–0.037).

**Conclusions:**

In a homogeneous group of young to middle-aged healthy females aging had an effect on T2 and R2* relaxation times and former GBCA applications influenced the measured T1 relaxation times.

**Key Points:**

- The quantitative T1, T2, and R2* relaxation times measured in women at high risk of developing breast cancer showed characteristic bandwidth for all brain nuclei examined at 3.0-T MRI.
- The effect of participant age had a comparatively strong impact on R2*, T2, and T1 relaxation times for the majority of brain nuclei examined.
- *The effect of prior GBCA administrations on T1 relaxation times rates was comparatively less pronounced, yielding statistically significant results for the dentate nucleus, globus pallidus, and pons.*

**Summary statement:**

Healthy women with and without previous GBCA-enhanced breast MRI exhibited age-related T2* and T2 relaxation alterations at 3.0 T-brain MRI. T1 relaxation alterations due to prior GBCA administration were comparatively less pronounced.

**Supplementary Information:**

The online version contains supplementary material available at 10.1007/s00330-021-08069-4.

## Introduction

Although relaxation changes due to aging and ingestion of metalliferous foods are widely considered to be present in healthy adults, there is a paucity of data on typical visible changes on MR imaging [1–4]. Contrary to the majority of chemical elements normally ingested, the earth metal gadolinium (Gd) is not a physiologically inherent component of the human body [5–7]. Since their clinical introduction in 1988, gadolinium-based contrast agents (GBCAs) were considered to have an excellent safety profile with reported serious adverse reactions in the range of 0.03% [6, 8]. Since 2014, however, retrospective studies have indicated an association between previous GBCA administration and increased signal intensity, predominantly in the dentate nucleus and the globus pallidus, on unenhanced T1-weighted MR images [9, 10]. The observed signal intensity changes seemed dose dependent and to be associated more often with linear GBCAs than macrocyclic GBCAs in rodent and human studies [6, 9, 11–20]. Harmful side effects of Gd deposits in the brain have been discussed [21]. However, a recent population-based study found no association between prior Gd exposure and neurodegenerative disease [22]. Although the long-term clinical relevance of cerebral Gd-deposits currently remains unknown [7], for safety reasons, the use of linear GBCAs has been prohibited in the European Union since 2017/2018 [7, 23].

Due to their high paramagnetic properties, GBCAs are of pivotal importance for contrast-enhanced breast MRI which constitutes the most sensitive method for early breast cancer detection available [24–28]. Therefore, women with an increased genetic risk of developing breast cancer aged 30 to 50 years undergo annual GBCA-enhanced breast MR imaging like in most international intensified screening programs [26, 27]. This group thus represents a unique collective of healthy women inherently at risk of Gd exposure from GBCA administration.

Recently, a case-control study reported no GBCA-associated T1 signal increases in brain nuclei exposed to ≥ 6 GBCA doses and unexposed controls [29]. The study was limited by age differences between cases and controls and examined T1 relaxation only. The current investigation aims to extend this preliminary evidence and thus was based on a larger number of MRI examinations and a balanced case-control population regarding participants’ age. We pursued the working hypothesis:

- That it is possible to generate quantitative T1, T2, and R2*(1/T2*) acquisition sequences with a spatial and contrast resolution suitable for detecting GBCA- and age-associated relaxation time differences in brain nuclei and
- That we succeed in demonstrating possible age- and dose-dependent relaxation time changes and signal intensity changes in brain nuclei following multiple intravenous doses of GBCA compared to Gd-naïve controls.

## Material and methods

### Study participants

This single-center, investigator-initiated, prospective case-control study was carried out with the approval of the institutional ethics review board (file reference 16-240) and was not supported by industrial sponsoring.

**Fig. 1:**
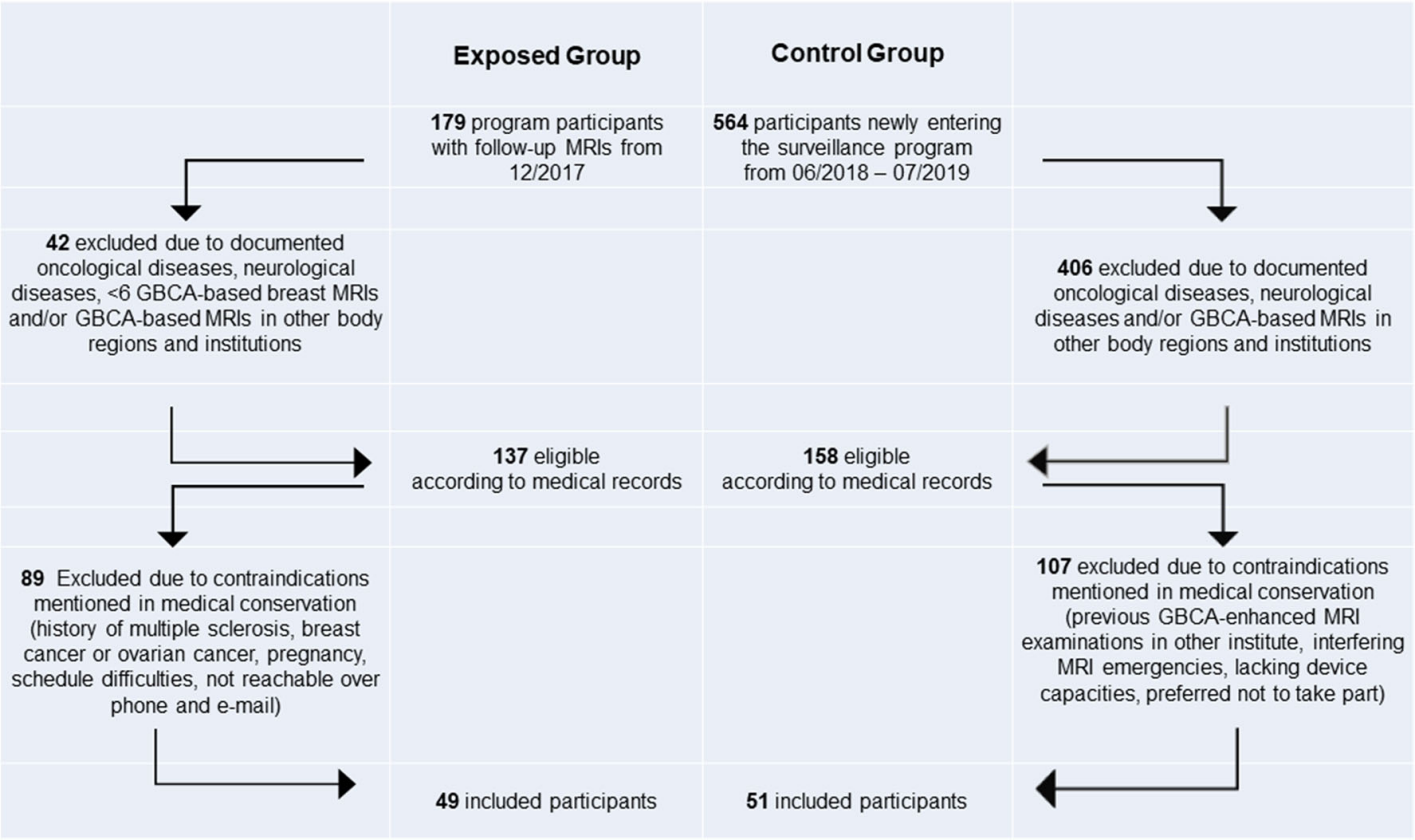
Recruitment of the participants taking part in the exposed group and in the control group

All women included were attenders of the local center of the National Intensified Early Breast Cancer Detection Program. They were at high risk of developing breast cancer, had no history of cancer or neurological disease, and had provided written informed consent to take part in the study. The women in the exposed group had previously received at least six doses of macrocyclic GBCA in the context of surveillance. The threshold of 6 GBCA-guided breast MRI examinations was chosen as trade-off between the increasing likelihood of Gd retention with higher numbers of GBCA exposures and the number of healthy advice-seekers at our family breast center with higher numbers of GBCA applications. The control group comprised women newly entering the program with no prior GBCA exposure and were prospectively included during a period of 11 months. All women eligible for study participation were identified via the comprehensive electronic Hospital and Radiology Information Systems of the University Hospital (ORBIS® OpenMed/RIS Nice®, Dedalus Healthcare Systems Group) (Fig. 1). Inclusion and exclusion criteria were confirmed via telephone calls and on-site consultations (B.K.). The members of the exposed group had never received any other contrast medium other than gadoterate meglumine and the controls had never received any GBCA according to their own account and all available medical data. Eligible participants with uncertain medical history, a history of cancer or neurological disease, compromised kidney function, and/or < 6 contrast-based breast MRIs, contrast-based MRIs of other body regions (exposed group), or any GBCA-enhanced MRIs (controls) were excluded.

The breast MRI protocols used were in accordance with international recommendations [30]. Gadoterate meglumine (Dotarem®, Guerbet GmbH) was the only contrast agent used at our institution since 2006. The contrast medium was injected via a cubital vein at a dose of 0.1 mmol/kg body weight. The total number and cumulative volume of GBCA administered were obtained from the electronic Radiology Information System.

### MR imaging

All participants underwent unenhanced MR imaging of the brain using a clinical 3.0-T scanner (Ingenia 3, Philips Healthcare) and a vendor-supplied 16-channel head coil. The median time interval between the last GBCA-based breast MRI and the native brain MRI examination was 89 days (range 18 to 254 days). The participants were positioned head first in supine position in the MRI scanner. Image data acquisition was in axial plane. It included T1 mapping for the quantification of T1 relaxation using a 2D Inversion Recovery Look-Locker (LL) sequence and a B1-corrected 3D Variable Flip-Angle (VFA) Gradient Echo sequence, T2 mapping for the quantification of T2 relaxation using a 2D Multi Gradient Spin Echo sequence, and R2* mapping for R2* quantification using a 2D Multi Gradient Echo sequence (Table 1). Furthermore, T1-weighted 2D Spin Echo (T1wSEM), T1-weighted 3D Turbo Gradient Echo (T1w3D), and T2-weighted 2D Turbo Spin Echo sequences (T2wTSE) were carried out in order to obtain T1- and T2-weighted images.

**Table 1:** Data acquisition parameters. For T2 mapping, the number of spin echo/gradient echoes is reported

Name	Look-Locker T1 mapping	Variable FA T1 mapping	T2 mapping	R2 star mapping	T1w 3D (MPRAGE)	T1w SEM	T2w TSE
Type	2D Look-Locker T1 mapping	3D variable flip angle TFE	2D multi-gradient spin echo	2D multi-gradient echo	3D turbo gradient echo	2D spin echo	2D turbo spin echo
Weighting	T1 quantification	T1 quantification	T2 quantification	R2* quantification	T1	T1	T2
Repetition time, TR (ms)	3.7	9	3695	24	7.9	705	3000
Number of echoes	-	-	5/16	16	-	-	-
Shot repetition time (ms)	8000	-	-	-	2100	-	-
Inversion times (ms)	160, 760, …, 3760	-	-	-	950	-	-
Echo time TE (ms)	1.07	3.7	20, 40, …, 320	1.92, 3.32, …, 22.92	3.5	13	80
Acquisition matrix	152 × 121	240 × 220	256 × 160	192 × 153	252 × 200	256 × 204	420 × 270
Field of view, FOV (mm × mm × mm)	230 × 180 × 25	240 × 220 × 160	230 × 182 × 120	230 × 183 × 120	249 × 200 × 170	250 × 187 × 125	231 × 180 × 112
Slice thickness (mm)	5	1	2	4	1	5	4
Acquisition time (min)	03:40	05:23	04:18	00:59	04:41	02:26	01:54

### Image analysis

**Fig. 2:**
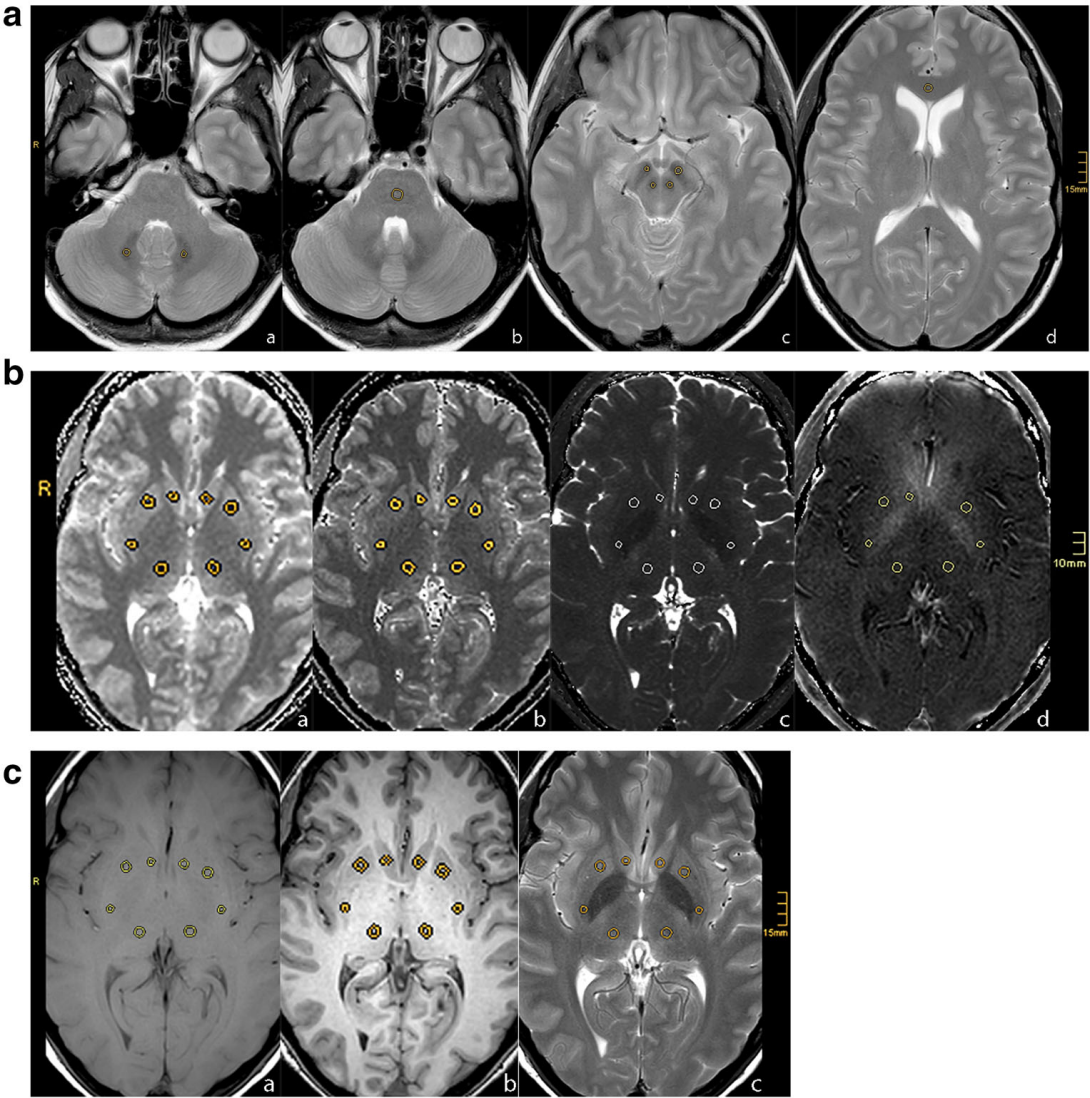
Anatomy-based placement of regions-of-interests (ROI) in a 48-year-old study participant who had received 13 doses of gadolinium-based contrast agents (GBCA) with a cumulative volume of 194 mL in the last 9 years prior to the target brain MRI. **a**: ROI placements in the dentate nucleus (**a**), the pons (**b**), the substantia nigra and the nucleus ruber (**c**), and in the crus anterior of the internal capsule (**d**) in the T2wTSE acquisition sequence. **b**: ROI placements in the globus palidus, the putamen, the caudate nucleus, and the thalamus for the acquisition sequences Look-Locker (LL) T1 mapping (**a**), variable flip angle (VFA) T1 mapping (**b**), T2 mapping (**c**), and R2* mapping (**d**). **c**: ROI placements in the globus palidus, the putamen, the caudate nucleus, and the thalamus for the qualitative T1w3D sequence (**a**), the qualitative T1wSEM (**b**), and the qualitative T2wTSE sequence

Image data were pseudonymized and stored as uncompressed DICOM files in PACS (ImpaxEE®, Dedalus Healthcare Systems Group). Image data were analyzed using a dedicated PACS-workstation. The region of interest (ROI) was initially placed by one investigator (B.K.) and subsequently verified by a board-certified neuroradiologist (M.S.), both with > 20 years of experience in neuroradiology. The initial placement of the ROI was adjusted in only 6 cases, thus indicating high interrater agreement. Both investigators mutually marked the target regions dentate nucleus (DN), pons (PO), caudate nucleus (CN), crus anterior (CA) of the capsula interna, globus pallidus (GP), nucleus ruber (NR), putamen (PU), substantia nigra (SN), and thalamus (TH) on the T2-weighted images. The interactively defined regions of interest (ROI) on the T2-weighted images were automatically copied onto the corresponding images of the other series (Fig. 2). Bilateral target nuclei were evaluated individually. Anatomical incongruities due to slight body movements during the examinations were adjusted by manual ROI corrections. The average relaxation times and signal intensities were read out of these ROIs.

### Statistical analysis

The relaxation times derived from the quantitative measurements and the signal intensities derived from the qualitative acquisition series were digitally documented (Excel®, Microsoft Corp.). The statistical calculations were carried out using SPSS Statistics software (IBM Corp.). All variables were summarized by mean, standard deviation (SD), minimum (min), and maximum (max) values. Measurements derived from bilateral brain nuclei were averaged. The relaxation time/signal intensity ratios of supratentorial brain nuclei to the CA and infratentorially the DN to the PO were calculated. No ratios were given for CA and pons in the numerator because the measurement results of both brain nuclei are given in the denominator of the corresponding ratios of other deep brain nuclei. Spearman rho correlations coefficients was used to assess the (pairwise) relationship between participant age, number/cumulative volume of GBCA-administration, and relaxation time/signal intensity (ratio) because the variables were not normally distributed. Multivariable linear regressions were performed to adjust for the possible effect of the variable age. Collinearity was checked by calculating variance inflation factors. Consequently, we abstained from including both the number and the cumulative volume of GBCA administrations as covariates. The dependency of relaxation time/intensity (ratios) and the time span between the last GBCA-based breast MRI and the target brain examination were assessed likewise. Scatter plots were used for graphical illustration. All analyses were essentially explorative with *p* values ≤ 0.05 (*) indicating moderate evidence, *p* values ≤ 0.01 (**) intermediate evidence, and *p* values ≤ 0.001 (***) strong evidence against the null hypothesis (e.g., zero correlation).

## Results

### Study participants

The study group consisted of 100 women with a median age of 41.5 years (minimum 24 years, maximum 63 years) (Table 2). The age distribution did not differ between both groups (exposed: 42 (24 – 63) years, controls: 38 (28 – 57) years). The exposed participants had received a median of 8.0 doses (range: 6 to 14 doses) of gadoterate meglumine prior to the target brain MRI, the median cumulative GBCA dose being 119 mL (69 to 194 mL). The mean time interval between the last GBCA-enhanced breast MRI and the target brain MRI was 104 ± 55 days (18 to 254 days). Diagnostic assessment of the acquired brain MRI images yielded normal results in all study examinations.

**Table 2:** Participant demographics. All participants were female. Data are given as numbers (n), mean ± standard deviation or median and range. *GBCA* gadolinium-based contrast agent, *y* years

Parameter	Value
Total no. of participants	100
Mean age (y)	41.5 (24 – 63)
Participants without GBCA exposure	51
Mean age (y)	42 (24 – 63)
Participants exposed to GBCA	49
Mean age (y)	38 (28 – 57)
Mean time interval since last GBCA exposure (days)	104 ± 55
Minimum and maximum time interval since last GBCA exposure (days)	18; 254
Median cumulative gadoterate meglumine doses (mL)	119 (69 – 194)
Median number of gadoterate meglumine doses	8.0 (6 – 14)

### Quantitative MRI data acquisitions

#### Relaxation times

The numeric differences between the participants exposed to ≥ 6 GBCA doses and the GBCA naïve controls were indiscernible on the MR images.

The quantitative relaxation times for all examined brain regions showed homogeneous distributions for all acquisition sequences in the examined brain nuclei (Table 3). The T1 relaxation times measured by LL mapping were consistently higher than those of VFA mapping and differed systematically in the same direction (range: ΔCA 161 ms to ΔCN 333 ms). The shortest T1 relaxation was measured in the CA (794 ± 48 ms/948 ± 51 ms) and the longest in the CN (1279 ± 64 ms/16281 ± 95 ms).

**Table 3:** T1 relaxation times (ms), T2 relaxation times (ms), and R2* relaxation rates (1/s) listed according to age groups and exposed participants vs. controls. The LL mapping sequences did not cover the DN and PO due to the limited coverage in the z-direction

					Look-Locker (LL) T1 mapping			Variable flip-angle (VAF) T1 mapping			T2 mapping			R2* mapping		
					Valid N	Mean	Standard deviation	Valid N	Mean	Standard deviation	Valid N	Mean	Standard deviation	Valid N	Mean	Standard deviation
CA	Age (years)	Up to 40	Group	Control	21	785	43	26	940	53	29	68	5	29	34	6
				Exposed	11	773	34	12	948	53	12	67	3	12	31	8
		40 to 50	Group	Control	7	796	33	8	943	38	8	69	5	8	32	6
				Exposed	27	776	35	29	944	53	29	68	3	29	36	9
		Over 50	Group	Control	12	808	62	13	949	63	14	70	4	14	31	8
				Exposed	7	790	24	7	967	42	7	69	4	7	32	7
		Total	Group	Control	40	794	48	47	943	53	51	69	5	51	33	7
				Exposed	45	778	33	48	948	51	48	68	3	48	34	9
CN	Age (years)	Up to 40	Group	Control	28	1288	61	26	1588	75	29	77	5	29	32	9
				Exposed	11	1284	57	12	1637	122	12	77	4	12	36	6
		40 to 50	Group	Control	8	1296	52	8	1575	60	8	76	3	8	38	8
				Exposed	26	1275	44	29	1613	65	29	74	4	29	36	9
		Over 50	Group	Control	14	1251	72	13	1618	103	14	75	5	14	33	5
				Exposed	7	1267	42	7	1672	142	7	74	3	7	35	15
		Total	Group	Control	50	1279	64	47	1594	81	51	76	4	51	33	8
				Exposed	44	1276	47	48	1628	95	48	75	4	48	36	9
DN	Age (years)	Up to 40	Group	Control				26	1099	48	29	61	5	29	36	5
				Exposed				12	1074	43	12	61	6	12	34	6
		40 to 50	Group	Control				8	1096	86	8	59	7	8	39	6
				Exposed				29	1074	54	29	60	6	29	38	7
		Over 50	Group	Control				13	1093	76	14	57	8	14	41	7
				Exposed				7	1053	45	7	59	8	7	39	8
		Total	Group	Control				47	1097	62	51	60	6	51	38	6
				Exposed				48	1071	50	48	60	7	48	37	7
GP	Age (years)	Up to 40	Group	Control	28	1062	48	26	1277	67	29	58	6	29	27	4
				Exposed	11	1038	31	12	1272	71	12	56	4	12	28	3
		40 to 50	Group	Control	8	1023	51	8	1229	71	8	55	4	8	32	5
				Exposed	26	1002	40	29	1245	53	29	54	5	29	32	6
		Over 50	Group	Control	14	1019	56	13	1269	79	14	55	7	14	33	7
				Exposed	7	1039	51	7	1260	57	7	53	12	7	35	13
		Total	Group	Control	50	1044	54	47	1267	72	51	57	6	51	29	6
				Exposed	44	1017	43	48	1254	58	48	54	6	48	31	7
NR	Age (years)	Up to 40	Group	Control	13	865	37	26	1040	50	29	61	7	29	32	4
				Exposed	8	864	31	12	1049	50	12	60	4	12	32	4
		40 to 50	Group	Control	2	842	37	8	1009	41	8	58	4	8	34	4
				Exposed	21	849	53	29	1039	52	29	57	4	29	35	5
		Over 50	Group	Control	9	838	50	13	1024	60	14	60	9	14	35	6
				Exposed	7	855	28	7	1042	60	7	57	5	7	37	3
		Total	Group	Control	24	853	43	47	1030	52	51	60	7	51	33	5
				Exposed	36	854	44	48	1042	52	48	58	4	48	34	**4**
PO	Age (years)	Up to 40	Group	Control				26	1201	53	29	82	7	29	27	10
				Exposed				12	1168	78	12	84	14	12	31	15
		40 to 50	Group	Control				8	1183	75	8	84	6	8	29	11
				Exposed				29	1131	62	29	80	7	29	31	12
		Over 50	Group	Control				13	1152	52	14	82	8	14	25	5
				Exposed				7	1187	90	7	83	7	7	25	4
		Total	Group	Control				47	1184	60	51	82	7	51	27	9
				Exposed				48	1149	72	48	82	9	48	30	12
PU	Age (years)	Up to 40	Group	Control	28	1214	48	26	1390	99	29	65	6	29	27	8
				Exposed	11	1202	49	12	1407	114	12	65	6	12	27	5
		40 to 50	Group	Control	8	1189	62	8	1392	70	8	63	3	8	28	4
				Exposed	27	1197	31	29	1413	106	29	63	6	29	29	5
		Over 50	Group	Control	14	1184	65	13	1402	99	14	64	4	14	28	4
				Exposed	7	1195	45	7	1440	83	7	63	3	7	29	4
		Total	Group	Control	50	1202	56	47	1393	93	51	65	5	51	27	6
				Exposed	45	1198	38	48	1416	103	48	63	6	48	28	5
SN	Age (years)	Up to 40	Group	Control	13	848	35	26	1067	70	29	56	7	29	35	7
				Exposed	8	836	25	12	1053	67	12	51	5	12	38	6
		40 to 50	Group	Control	2	882	19	8	1059	83	8	52	4	8	38	7
				Exposed	21	826	34	29	1030	63	29	54	6	29	37	5
		Over 50	Group	Control	9	829	60	13	1001	56	14	51	5	14	40	6
				Exposed	7	835	32	7	1042	69	7	53	4	7	37	3
		Total	Group	Control	24	844	46	47	1047	73	51	54	6	51	37	7
				Exposed	36	830	31	48	1038	64	48	53	6	48	38	5
TH	Age (years)	Up to 40	Group	Control	28	923	47	26	1184	72	29	73	4	29	23	1
				Exposed	11	940	49	12	1198	72	12	72	5	12	22	1
		40 to 50	Group	Control	8	936	53	8	1190	47	8	73	2	8	23	1
				Exposed	26	933	47	29	1183	46	29	71	4	29	23	1
		Over 50	Group	Control	14	946	48	13	1220	83	14	75	3	14	23	1
				Exposed	7	945	35	7	1195	62	7	75	2	7	22	2
		Total	Group	Control	50	932	48	47	1195	72	51	73	4	51	23	1
				Exposed	44	937	45	48	1188	55	48	72	4	48	23	1
				Total	94	934	47	95	1192	64	99	73	4	99	23	1

#### Univariable comparisons

**Fig. 3:**
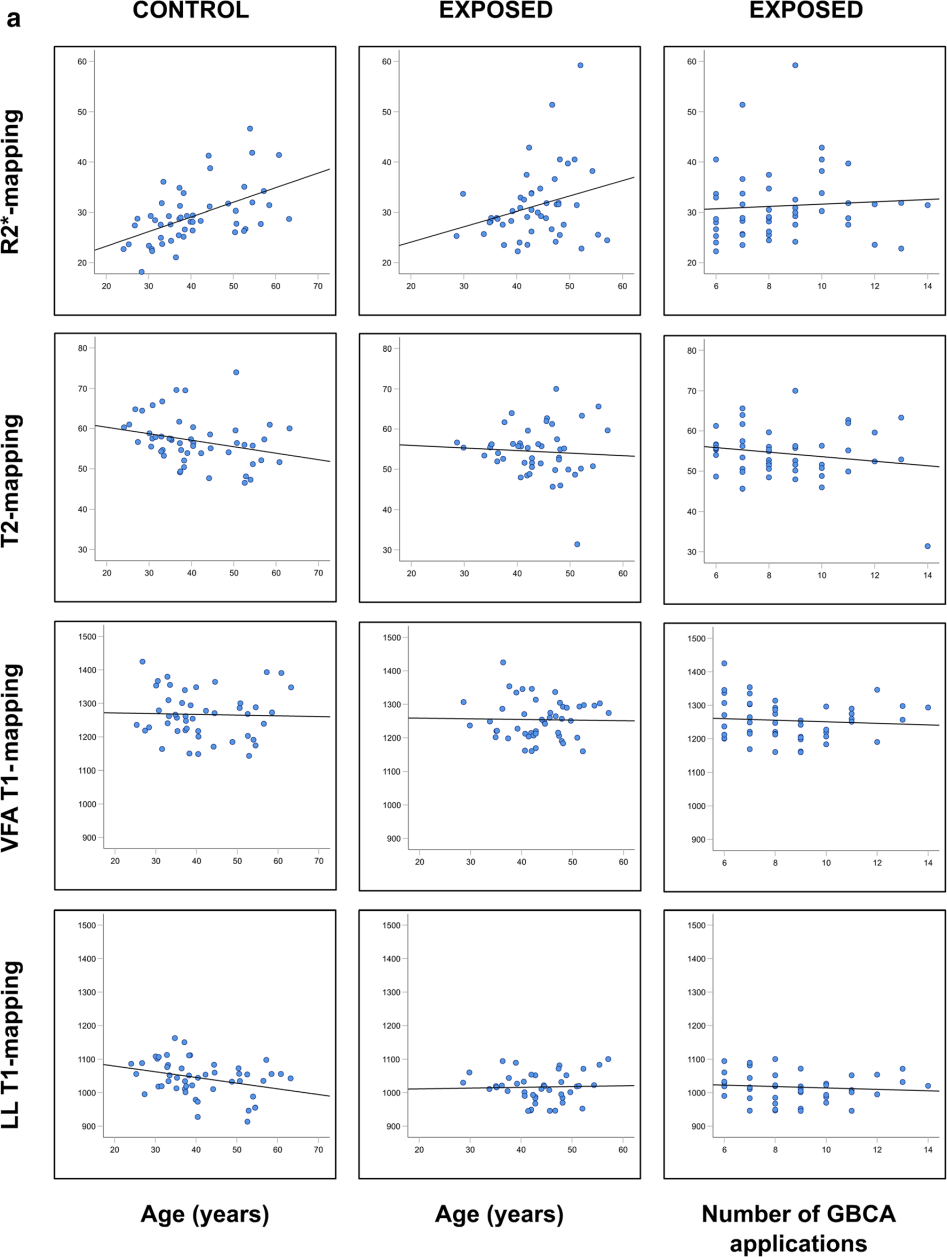

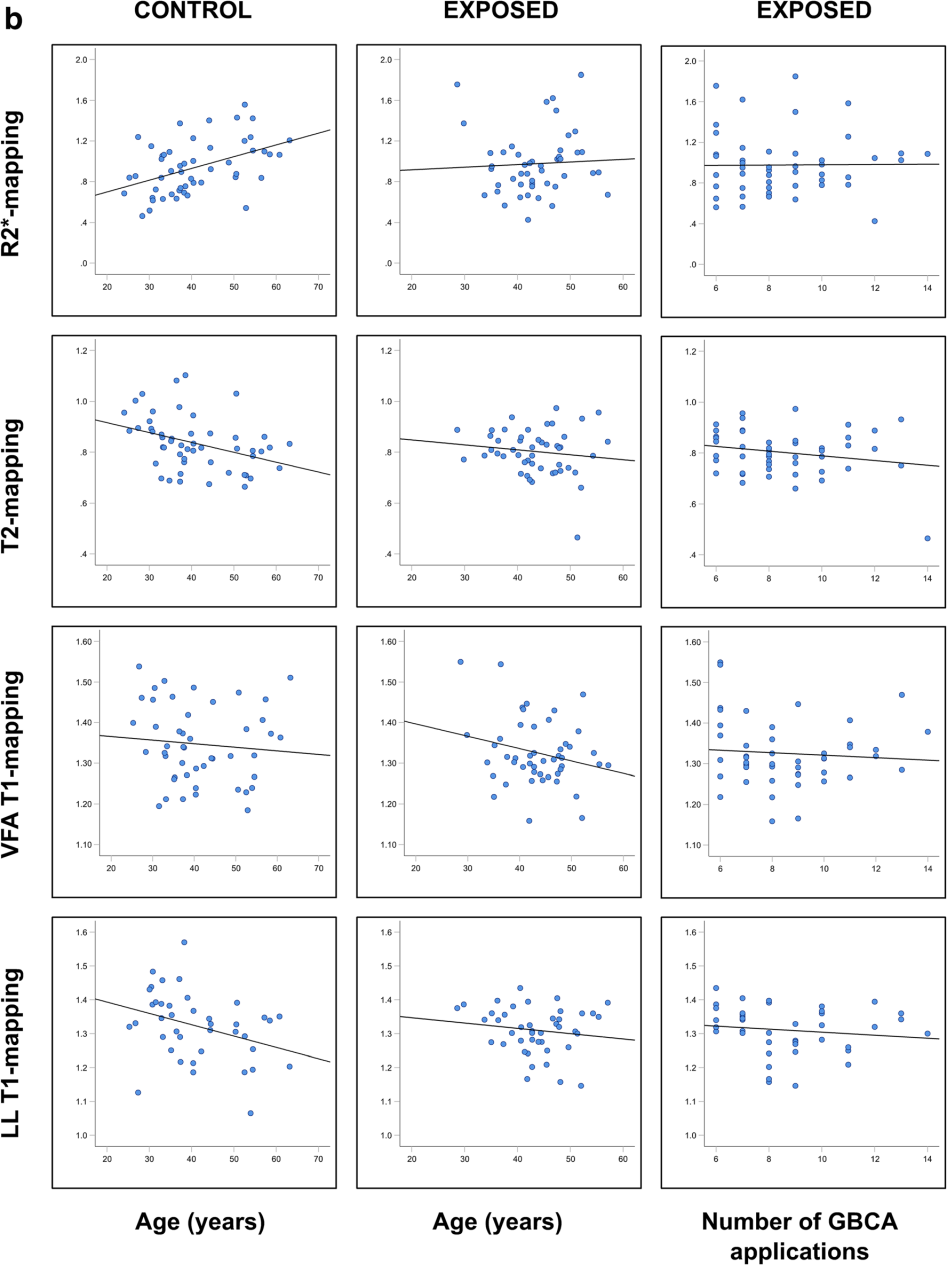

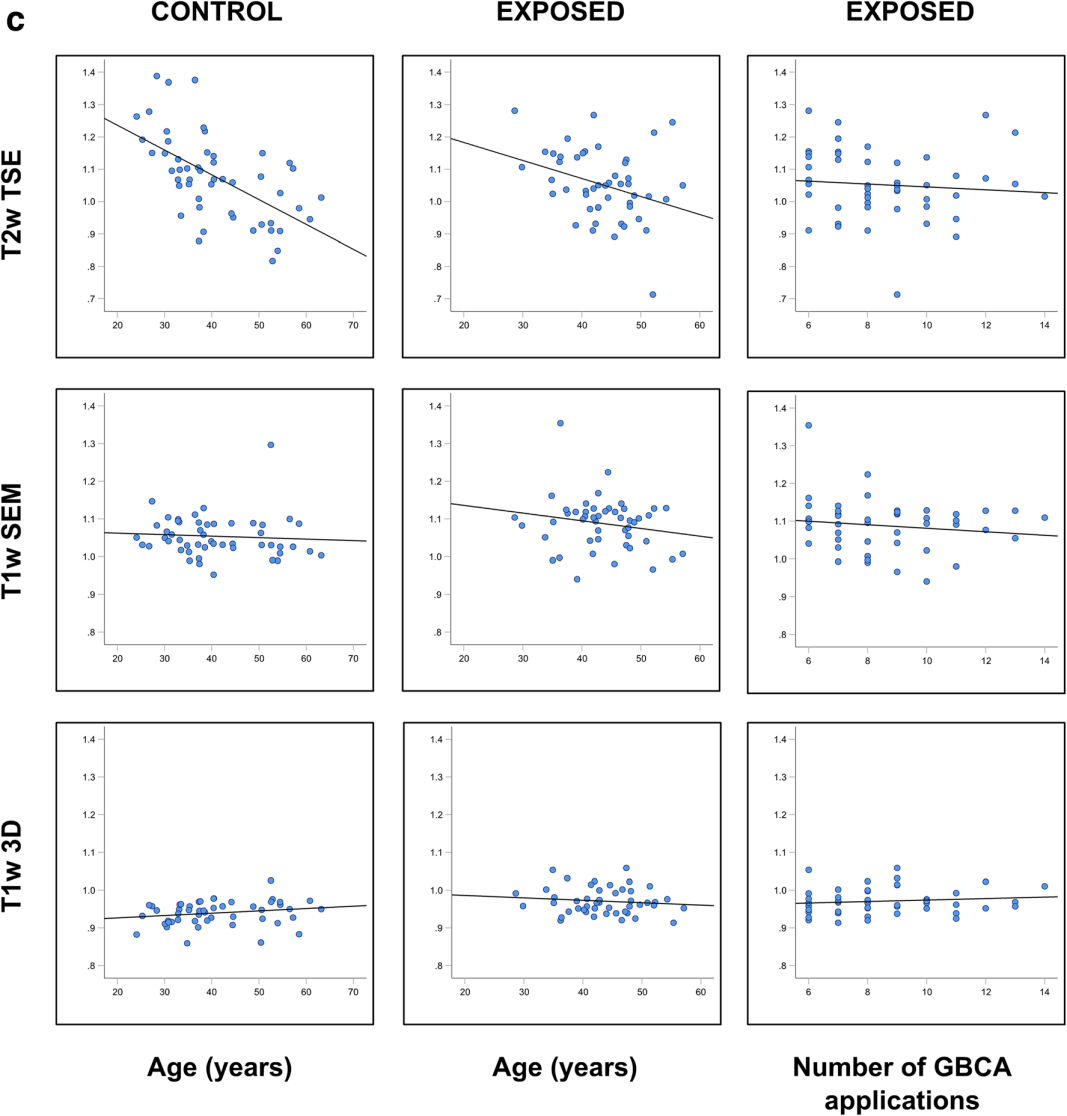
Interdependency of age (left and middle columns) and the number of prior GBCA doses (right column) and the relaxation time(s)/signal intensity ratios measured in the globus pallidus (GP) and the crus anterior (CA) of the internal capsule. **a**: R2* mapping, T2 mapping, VAFT1 mapping, and LL-T1 mapping in the GP. **b**: Ratios GP:CA for R2* mapping, T2 mapping, VFA 1 mapping and LL-T1 mapping. **c**: Ratios GP:CA for the qualitative T1-weighted and T2-weighted data acquisition sequences

The effect variable “age” revealed negative correlation coefficients with increasing age for T1 mapping in both groups and for the majority of brain nuclei reaching moderate significance in the controls in the GP (*p* = 0.025), PO (*p* = 0.025), and SN (*p* = 0.013) (Table 4, Fig. 3a). The correlation coefficients for “age” were also mostly negative in T2 mapping reaching moderate to high significance in the GP and the SN (controls: *p* = 0.047 and 0.006) and NR (exposed: *p* = 0.039). Due to the inverse read out compared to the T2* relaxation times, the R2* correlation coefficients were mostly positive reaching moderate to high significance in the control group (DN *p* < 0.000, NR *p* = 0.020, GP *p* < 0.000, SN *p* < 0.021) and in the exposed group (DN *p* = 0.009, NR *p* = 0.033).

**Table 4:** Univariable analyses comparing the correlation coefficients of the measured relaxation times and signal intensity ratios for the confounders “age,” “decay,” “number of GBCA dosages,” and “cumulative volume of GBCA dosages.” No ratios were given for the CA and pons in the numerator because the measurement results of both brain nuclei are given in the denominator of the corresponding ratios of other deep brain nuclei

Target region		Control group				Exposed group					Control group			Exposed group		
	Confounder	LL-T1 mapping	VFA-T1 mapping	T2 mapping	R2* mapping	LL-T1 mapping	VFA-T1 mapping	T2 mapping	R2* mapping	Ratio	T1w3D	T1w SEM	T2w TSE	T1w3D	T1w SEM	T2w TSE
CA	Age	0.109	0.054	0.326*	−0.122	0.240	0.196	0.109	−0.026							
	Decay					−0.236	−0.135	−0.082	0.132							
	Number					0.025	−0.013	0.052	0.069							
	Volume					0.098	−0.049	0.033	−0.066							
CN	Age	−0.157	0.102	−0.196	0.279^*^	−0.015	0.081	−0.182	−0.148	CN:CA	0.301^*^	−0.008	−0.238	0.040	−0.071	−0.236
	Decay					−0.024	0.152	−0.039	0.189					−0.022	0.096	0.197
	Number					−0.066	−0.142	−0.159	−0.055					−0.086	0.015	−0.151
	Volume					−0.171	−0.216	−0.153	−0.102					0.050	0.056	−0.123
DN	Age		−0.126	−0.191	0.365^**^		−0.089	−0.074	0.338^*^	DN:PO	0.035	−0.112	−0.251	−0.074	−0.168	−0.020
	Decay						−0.202	−0.084	0.355^*^					−0.122	−0.031	−0.090
	Number						−0.078	0.060	0.203					0.105	−0.102	0.159
	Volume						−0.092	−0.043	0.194					0.057	−0.107	0.075
GP	Age	−0.322*	−0.080	−0.368*	0.535***	0.004	0.025	−0.134	0.253	GP:CA	0.278^*^	−0.238	−0.588^**^	−0.065	−0.116	−0.319^*^
	Decay					−0.260	−0.103	−0.202	0.320^*^					−0.006	0.013	−0.137
	Number					−0.162	−0.122	−0.141	0.173					0.140	−0.101	−0.167
	Volume					−0.134	−0.037	−0.070	0.176					0.236	0.062	−0.187
NR	Age	−0.117	−0.119	−0.275	0.315^*^	0.045	−0.007	−0.307^*^	0.375^**^	NR:CA	0.079	−0.066	−0.437^**^	0.093	−0.212	−0.267
	Decay					0.158	−0.172	−0.116^*^	0.075^**^					−0.110	−0.032	−0.095
	Number					0.079	−0.110	−0.147	0.140					−0.057	−0.155	−0.293
	Volume					0.104	0.000	−0.181	0.110					0.195	−0.024	−0.193
PO	Age		−0.318^*^	0.135	0.035	0.546	−0.084	0.059	0.037							
	Decay						−0.260	0.124	0.065							
	Number						−0.017	0.093	0.234							
	Volume						−0.049	0.140	0.129							
PU	Age	−0.152	−0.016	−0.254	0.398^**^	−0.079	0.099	−0.237	0.169	PU:CA	0.143	−0.017	−0.326^*^	0.052	−0.094	−0.236
	Decay					−0.112	0.081	−0.049	0.266					0.024	0.081	0.177
	Number					−0.036	0.126	−0.208	0.209					0.124	0.072	−0.125
	Volume					0.041	0.221	−0.054	0.168					0.077	0.132	−0.016
SN	Age	−0.099	−0.313^*^	−0.429^**^	0.364^**^	0.042	−0.140	0.124	0.084	SN:CA	0.056	−0.127	−0.541^**^	0.104	−0.176	−0.088
	Decay					−0.133	0.133	−0.190	0.151					−0.311^*^	−0.033	−0.242
	Number					−0.178	−0.016	0.116	−0.054					−0.071	−0.127	−0.018
	Volume					−0.136	0.082	−0.030	−0.133					−0.042	0.029	−0.056
TH	Age	0.121	0.084	0.300^*^	−0.128	0.168	−0.057	0.336^*^	0.195	TH:CA	0.150	−0.032	−0.060	−0.022	−0.017	0.002
	Decay					0.080	0.086	−0.155	0.264					−0.107	0.022	0.215
	Number					0.234	0.155	0.202	0.026					−0.328^*^	0.009	0.235
	Volume					0.190	0.028	0.174	0.127					−0.050	0.104	0.174

Spearman rho correlations and significances are given as *p ≤ 0.05, **p ≤ 0.01, ***p < 0.001

The time interval between the last GBCA-based breast MRI and the target brain MRI (effect variable “decay”) negatively correlated with T1 relaxation times measured in most brain nuclei reaching moderate significance in the CA and GP (*p* = 0.032 and 0.040). The correlation of the effect variable “decay” and measured R2* relaxation values was mostly positive due to the sign inversion compared to T2* times showing moderate significance in the DN and GP (*p* = 0.040 and 0.03).

The collinear effect variables “number” and “cumulative volume” of applied GBCA doses correlated negatively with most of the measured T1 relaxation times without reaching statistical significance due to the shortening of T1 relaxation caused by GBCA. The T2 measurements showed indecisive results, whereas the correlation coefficients in R2* mapping were mostly positive without reaching statistical significance.

#### Multivariable analyses

Multivariable analysis of the effect variables “age” and “group comparison,” the latter defined as the comparison of the GBCA-exposed group and the GBCA-naïve controls, confirmed significant correlations for “age” in the GP (*p* = 0.005) and SN (*p* = 0.046) in T1 mapping and moderate significance in the CA (*p* = 0.038), CN (*p* = 0.024), NR (*p* = 0.048), SN (*p* = 0.048), and TH (*p* = 0.015) in T2 mapping (Table E1). “Age” had a highly significant influence on the relaxation rates in R2* mapping in the DN (*p* = 0.001), GP (*p* < 0.000), NR (*p* = 0.001), and SN (*p* = 0.02).

Regarding the effect variable “group comparison,” which reflects the overall difference between the exposed group and the control group and not just the impact of GBCA, multivariable analysis revealed negative orientations of the slope coefficients (B-values) correlating with a tendency towards a shortening of T1 relaxation times due to GBCA application. The observed negative correlations were moderately significant in the CA (*p* = 0.037) in LL mapping and in the DN (*p* = 0.033) and PO (*p* = 0.019) in VFA mapping. We did not notice any significant effect of the effect variable “group comparison” regarding T2 and R2* mapping.

These results were confirmed for “age” when normalizing the relaxation times for each participant by calculating intraindividual ratios between the relaxation times measured in the target brain nuclei and in the CA (supratentorial) and PO (infratentorial) (Table E1, Fig. 3b). The calculated slope coefficients were predominantly negative in T1/T2 mapping and positive in R2* mapping due to the inverse read out of the T2* signal. The influence of “age” on the relaxation time ratios showed moderate to intermediate significance for ratios SN:CA (*p* = 0.004), CN:CA (*p* = 0.021), NR:CA (*p* = 0.013), GP:CA (*p* = 0.010), and GP:TH (*p* = 0.031) in LL mapping, for the ratio CN:CA in in VFA mapping (*p* = 0.005), for the ratio PU:CA (*p* = 0.022), CN:CA (*p* = 0.003), NR:CA (*p* = 0.003), GP:CA (*p* = 0.004), GP:TH (*p* = 0.002), and SN:CA (*p* = 0.007) in T2 mapping and moderate to strong correlations for ratios DN:CA (*p* = 0.018), GP:CA (*p* = 0.007), and GP:TH (*p* < 0.001) in R2* mapping.

The negative slope coefficients observed for the “number of GBCA applications” in the CA (*p* = 0.036), DN (*p* = 0.040), GP (*p* = 0.024), and PO (*p* = 0.026) (Table E2) and for the collinear effective variable “cumulative volume of applied GBCA doses” in the GP (*p* = 0.024) and PO (*p* = 0.026) reflect a shortening of T1 times with increasing numbers of applied GBCA doses per exposed study participant (Table E3). Likewise, the ratios of the relaxation times showed negative B-values in T1/T2 mapping and positive B-values in R2* mapping. The variable “age” had a moderate to intermediate influence on the ratios CN:CA (*p* = 0.019), NR:PO (*p* = 0.007), GP:CA (*p* = 0.012), and SN:CA (*p* = 0.003) in LL mapping and an intermediate level of significance for the ratio SN:CA (*p* = 0.006) in VFA mapping when regarding the number of applied GBCA doses. Correspondingly, the assessment of the effect variables “age” and “cumulative volume” of applied GBCA doses yielded correlations of moderate to intermediate significance for the ratios CN:CA (*p* = 0.020), NR:PO (*p* = 0.008), GP:CA (*p* = 0.013), GP:TH (*p* = 0.047), and SN:CA (*p* = 0.003). The influence of the effect variable “age” on the collinear effect variables “number” and “cumulative volume” of applied GBCA doses reached moderate significance for PU:CA (*p* = 0.025 and 0.022), CN:CA (*p* = 0.003 and 0.004), NR:PO (*p* = 0.004 and 0.005), GP:CA (*p* = 0.006 and 0.006), GP:TH (*p* = 0.003 and 0.003), and SN:CA (*p* = 0.005 and 0.006) in T2 mapping and moderate to strong significance for DN:PO (*p* = 0.010 and 0.012), GP:CA (*p* = 0.007 and 0.008), and GP:TH (*p* < 0.000 and < 0.000) in R2* mapping (Tables E2 and E3).

### Qualitative MRI data acquisitions

Univariable and multivariable analyses of the T1- and T2-weighted qualitative acquisition sequences for signal intensity essentially confirmed the aforementioned quantitative results (Tables 4 and Table E1). Multivariable analysis of the effect variables “age” and “group comparison” confirmed a significant correlation between “age” and the T2-weighted signal intensity ratios CN:CA (*p* = 0.003), NR:CA (*p* < 0.001), GP:CA (*p* < 0.001), GP:Th (*p* < 0.001), PU:CA (*p* < 0.001), and SN:CA (*p* < 0.001) (Table E1, Fig. 3c). In both T1-weighted sequences, the effect variable “group comparison” revealed moderate to strong correlations for the ratios CN:CA (*p* = 0.031 and 0.004), GP:CA (*p* < 0.001 and 0.005), and SN:CA (*p* = 0.010 and 0.046). The number and cumulative volume of applied GBCA doses showed similar results (Tables E2 and E3).

## Discussion

Our observation that the effect of aging on relaxation times measured in brain nuclei of young to middle-aged healthy females was predominantly seen as an age-related decrease in T2* relaxation time (DN, GP, NR: *p* ≤ 0.001) and in T2 relaxation time (CN, NR, SN: *p* = 0.015–0.048) and an only moderate decrease in T1 relaxation time for the GP (*p* = 0.046) is in accordance with preclinical studies, which have shown age-related concentration changes of iron, copper, and zinc in brain nuclei as well as associated microglial and astrocyte alterations [1–4]. Contrary to iron, copper, and zinc, the earth metal Gd is not a physiologically inherent component of the human body [5–7]. Because of its toxicity, Gd must be bound to a ligand in order to render it safe to be administered intravenously while maintaining its paramagnetic properties for MRI [7, 30]. Although it is known that a small fraction of intravenously applied GBCA is chronically retained in human tissues [14], little is known about its biochemical transformation, handling, and excretion [6, 7]. Histological studies have shown evidence of Gd deposition in brain tissue of patients with normal renal function [14, 17, 18, 31]. Spheroid Gd deposits were demonstrated in the basal lamina of cerebral microvessels and in the perivascular Virchow-Robin spaces of rodents after repeated injections of linear GBCAs [32]. The mechanism for deposition has not yet been fully elucidated and the clinical significance remains unclear [31, 33, 34].

Our results regarding quantitative T1 mapping with a 3.0-T MRI system are in accordance with the findings of Saake et al who, using a 1.5-T scanner, investigated 160 patients with multiple GBCA administrations and 60 GBCA-naïve control subjects and found significantly shortened T1 relaxation times in the GP in the exposed group [34]. Our findings also confirm those of Quattrocchi et al [24], who assessed the effect of age and number of previous injections of linear GBCA on signal intensity of unenhanced T1-weighted images of the DN and GP in 892 patients with prior GBCA exposure and 1906 subjects without. The results revealed a correlation of the signal intensity ratios DN:PO and GP:TH with age and the number of macrocyclic GBCA injections.

In the current study, quantitative T1/T2 mapping and qualitative T1/T2-weighted imaging were employed in order to investigate the effects of aging and Gd deposits on relaxation time. While quantitative relaxation values provide absolute measures and thus should allow for direct interindividual comparisons, they also imply the challenge of careful interpretation due to technical dependencies, which may have an impact on the quantification of results. Two different sequence designs were used for quantitative T1 mapping. The systematically higher relaxation time values in VFA mapping are explained by the differences in both sequence design, regarding spatial resolution and contrast resolution, and the number of T1 relaxation points assessed. T1 relaxation was read out at five points in LL mapping, whereas only two T1 relaxation read outs were carried out during the VFA acquisitions in order to gain a sufficient compromise between spatial resolution, contrast resolution, and the length of examination time. The presented ratio approach for the qualitative and quantitative measurements was chosen in order to ameliorate the influence of interindividual variances due to the individual concentrations of physiologically ingested metal ions like copper and iron in the brain nuclei examined, which cannot be quantified from patient history like the case with prior GBCA dosing.

The main limitation of the presented study lies in the interindividual comparison of healthy young women of the same age group. The lack of an intraindividual approach will be addressed in an ongoing study including attenders of the screening program. Further limitations may be seen in the small number of cases and the monocentric study design, as well as in the yet informal evaluation of interrater agreement on the basis of the ROI placements.

In conclusion, the known effects of aging and Gd exposure could be observed in the brain nuclei of a clinically homogeneous group of female adults using quantitative and qualitative 3.0-T MR imaging. The effect of aging was most pronounced in T2 and T2* MR imaging. The influence of prior GBCA applications on the measured T1 relaxation times and T1-weighted signal intensities were too small to be visually perceived on the MR images of brain nuclei.

## Supplementary Information

ESM 1 (DOCX 64.8 kb)

## Abbreviations

CA: Crus anterior of the internal capsule
CN: Caudate nucleus
DN: Dentate nucleus
GBCA: Gadolinium-based contrast agents
Gd: Gadolinium
GP: Globus pallidus
LL: Look-Locker
NR: Nucleus ruber
PO: Pons
PU: Putamen
SN: Substantia nigra
TH: Thalamus
VFA: Variable flip-angle

## Declarations

### Guarantor

The scientific guarantor of this publication is Prof. Dr. Med. Kathrin Barbara Krug.

### Statistics and biometry

Prof. Martin Hellmich, one of the authors of the manuscript, kindly provided statistical advice for this manuscript and carried out the biometric evaluations.

### Methodology

• Prospective

• Diagnostic or prognostic study

• Performed at one institution
