# Supplementary material for: Influence of aging and gadolinium exposure on T1, T2, and T2*-relaxation in healthy women with an increased risk of breast cancer with and without prior exposure to gadoterate meglumine at 3.0-T brain MR imaging
Source: Eur Radiol. 2021 Jul 3;32(1):331–45. doi: 10.1007/s00330-021-08069-4 (PMC8660719; doi:10.1007/s00330-021-08069-4)
Supplement: Supplementary file 1 — (DOCX 64.8 kb) [file 330_2021_8069_MOESM1_ESM.docx]

| Table E1 | Multivariable analyses regarding the influence of the differences between the control group comparison and the exposed group comparison (confounder "group comparison comparison") vs. the age of the study participant on relation time constants and the ratios of the signal intensities. B = signal change per year. Sig. = significance. | | | | | | | | | | | | | | | | |
| --- | --- | --- | --- | --- | --- | --- | --- | --- | --- | --- | --- | --- | --- | --- | --- | --- | --- |
| **Sequence** | | **B** | **95% Confidence Interval** | | **Sig.** | **B** | **95% Confidence Interval** | | **Sig.** | **B** | **95% Confidence Interval** | | **Sig.** | **B** | **95% Confidence Interval** | | Sig. |
|  |  |  | **Lower Bound** | **Upper Bound** |  |  | **Lower Bound** | **Upper Bound** |  |  | **Lower Bound** | **Upper Bound** |  |  | **Lower Bound** | **Upper Bound** |  |
| **Nucleus/ Ratio** | **Confounder** | **Look-Locker T1 mapping** | | | | **Variable Flip-Angle T1 mapping** | | | | **T2 mapping** | | | | **R2* mapping** | | | |
| **CA** | Age | 0.934 | -0.105 | 1.973 | 0.078 | 0.870 | -0.404 | 2.143 | 0.178 | 0.101 | 0.006 | 0.197 | **0.038*** | -0.031 | -0.215 | 0.153 | 0.737 |
|  | Group comparison | -18.783 | -36.390 | 0.0005 and p = 0.032. respectively) | **0.037*** | 3.645 | -17.723 | 25.013 | 0.736 | -0.945 | -2.580 | 0.690 | 0.254 | 1.579 | -1.569 | 4.727 | 0.322 |
| **CN** | Age | -1.011 | -2.365 | 0.342 | 0.141 | 1.322 | -0.838 | 3.482 | 0.227 | -0.114 | -0.213 | -0.015 | **0.024*** | 0.112 | -0.093 | 0.318 | 0.279 |
|  | Group comparison | -0.017 | -23.315 | 23.280 | 0.999 | 30.646 | -5.593 | 66.886 | 0.096 | -0.953 | -2.646 | 0.740 | 0.267 | 2.688 | -0.821 | 6.197 | 0.132 |
| **DN** | Age |  |  |  |  | -0.270 | -1.656 | 1.117 | 0.700 | -0.115 | -0.266 | 0.037 | 0.136 | 0.256 | 0.104 | 0.407 | **0.001***** |
|  | Group comparison |  |  |  |  | -25.281 | -48.543 | -2.019 | **0.033*** | 0.147 | -2.436 | 2.731 | 0.910 | -1.259 | -3.855 | 1.336 | 0.338 |
| **GP** | Age | -1.186 | -2.349 | -0.023 | **0.046*** | -0.210 | -1.819 | 1.399 | 0.796 | -0.135 | -0.276 | 0.006 | 0.060 | 0.294 | 0.154 | 0.434 | **0.000***** |
|  | Group comparison | -23.925 | -43.953 | -3.898 | **0.020*** | -12.241 | -39.233 | 14.751 | 0.370 | -2.184 | -4.590 | 0.223 | 0.075 | 1.152 | -1.245 | 3.549 | 0.342 |
| **NR** | Age | -0.291 | -1.638 | 1.057 | 0.668 | -0.383 | -1.659 | 0.893 | 0.552 | -0.137 | -0.272 | -0.001 | **0.048*** | 0.187 | 0.081 | 0.293 | **0.001***** |
|  | Group comparison | 1.210 | -22.110 | 24.530 | 0.918 | 12.184 | -9.224 | 33.592 | 0.261 | -2.297 | -4.615 | 0.020 | 0.052 | 0.946 | -0.860 | 2.752 | 0.301 |
| **PO** | Age |  |  |  |  | -1.406 | -3.015 | 0.203 | 0.086 | -0.008 | -0.198 | 0.182 | 0.932 | -0.066 | -0.319 | 0.187 | 0.606 |
|  | Group comparison |  |  |  |  | -32.584 | -59.585 | -5.583 | **0.019*** | -0.683 | -3.934 | 2.569 | 0.678 | 3.596 | -0.726 | 7.919 | 0.102 |
| **PU** | Age | -0.733 | -1.887 | 0.421 | 0.210 | 0.904 | -1.508 | 3.316 | 0.459 | -0.090 | -0.222 | 0.041 | 0.177 | 0.098 | -0.035 | 0.231 | 0.146 |
|  | Group comparison | -1.693 | -21.470 | 18.084 | 0.865 | 20.268 | -20.205 | 60.741 | 0.323 | -1.056 | -3.304 | 1.192 | 0.353 | 0.648 | -1.627 | 2.922 | 0.573 |
| **SN** | Age | -0.431 | -1.602 | 0.739 | 0.464 | -2.354 | -3.978 | -0.729 | **0.005**** | -0.142 | -0.282 | -0.002 | **0.048*** | 0.183 | 0.041 | 0.326 | **0.012*** |
|  | Group comparison | -13.036 | -33.290 | 7.217 | 0.203 | -4.229 | -31.484 | 23.027 | 0.759 | -0.248 | -2.645 | 2.149 | 0.838 | 0.129 | -2.309 | 2.567 | 0.917 |
| **TH** | Age | 0.677 | -0.452 | 1.805 | 0.237 | 0.503 | -1.073 | 2.079 | 0.528 | 0.116 | 0.023 | 0.209 | **0.015*** | -0.003 | -0.036 | 0.030 | 0.845 |
|  | Group comparison | 3.098 | -16.325 | 22.521 | 0.752 | -8.122 | -34.562 | 18.318 | 0.543 | -1.901 | -3.487 | -0.315 | 0.019 | -0.108 | -0.673 | 0.458 | 0.706 |
|  |  | **Look-Locker T1-mapping** | | | | **Variable Flip-Angle T1-mapping** | | | | **T2-mapping** | | | | **R2*-mapping** | | | |
| **CN:CA** | Age | -0.003 | -0.006 | 0.000 | **0.021*** | 0.000 | -0.003 | 0.003 | 0.845 | -0.003 | -0.005 | -0.001 | **0.003**** | 0.004 | -0.002 | 0.010 | 0.216 |
|  | Group comparison | 0.028 | -0.016 | 0.073 | 0.212 | 0.025 | -0.023 | 0.074 | 0.304 | -0.002 | -0.038 | 0.035 | 0.930 | 0.048 | -0.060 | 0.156 | 0.379 |
| **DN:PO** | Age |  |  |  |  | 0.001 | -0.001 | 0.003 | 0.315 | -0.002 | -0.003 | 0.000 | 0.105 | 0.011 | 0.002 | 0.021 | **0.018*** |
|  | Group comparison |  |  |  |  | 0.006 | -0.022 | 0.034 | 0.688 | 0.012 | -0.021 | 0.044 | 0.479 | -0.174 | -0.334 | -0.015 | **0.032*** |
| **NR:CA** | Age | -0.003 | -0.006 | -0.001 | **0.013*** | -0.002 | -0.003 | 0.000 | 0.131 | -0.003 | -0.006 | -0.001 | **0.003**** | 0.006 | 0.000 | 0.013 | 0.066 |
|  | Group comparison | 0.038 | -0.005 | 0.080 | 0.081 | 0.054 | -0.024 | 0.041 | 0.607 | -0.024 | -0.063 | 0.015 | 0.218 | 0.004 | -0.113 | 0.122 | 0.940 |
| **GP:CA** | Age | -0.003 | -0.005 | -0.001 | **0.010**** | -0.002 | -0.004 | 0.001 | 0.166 | -0.003 | -0.006 | -0.001 | **0.004**** | 0.009 | 0.003 | 0.016 | **0.007**** |
|  | Group comparison | -0.006 | -0.042 | 0.031 | 0.755 | -0.018 | -0.054 | 0.018 | 0.319 | -0.024 | -0.062 | 0.015 | 0.223 | 0.012 | -0.099 | 0.124 | 0.829 |
| **GP:TH** | Age | -0.002 | -0.004 | 0.000 | **0.031*** | -0.001 | -0.003 | 0.001 | 0.499 | -0.003 | -0.005 | -0.001 | **0.002**** | 0.013 | 0.007 | 0.019 | **0.000***** |
|  | Group comparison | -0.031 | -0.064 | 0.003 | 0.071 | -0.005 | -0.037 | 0.027 | 0.769 | -0.009 | -0.043 | 0.026 | 0.614 | 0.060 | -0.042 | 0.161 | 0.245 |
| **PU:CA** | Age | -0.002 | -0.005 | 0.000 | 0.069 | 0.000 | -0.004 | 0.003 | 0.785 | -0.003 | -0.005 | 0.000 | **0.022*** | 0.003 | -0.003 | 0.009 | 0.337 |
|  | Group comparison | 0.029 | -0.014 | 0.073 | 0.184 | 0.015 | -0.039 | 0.068 | 0.582 | -0.005 | -0.047 | 0.036 | 0.796 | 0.005 | -0.101 | 0.112 | 0.924 |
| **SN:CA** | Age | -0.003 | -0.005 | -0.001 | **0.004**** | -0.004 | -0.006 | -0.001 | **0.005**** | -0.003 | -0.006 | -0.001 | **0.007**** | 0.006 | -0.001 | 0.014 | 0.106 |
|  | Group comparison | 0.016 | -0.021 | 0.052 | 0.861 | -0.008 | -0.050 | 0.034 | 0.701 | 0.003 | -0.037 | 0.043 | 0.882 | -0.008 | -0.142 | 0.127 | 0.911 |
| **TH:CA** | Age | -0.001 | -0.003 | 0.001 | 0.469 | -0.001 | -0.003 | 0.002 | 0.639 | 0.000 | -0.002 | 0.002 | 0.989 | 0.000 | -0.005 | 0.005 | 0.924 |
|  | Group comparison | 0.026 | -0.011 | 0.063 | 0.163 | -0.014 | -0.054 | 0.027 | 0.506 | -0.016 | -0.048 | 0.015 | 0.311 | 0.041 | -0.090 | 0.073 | 0.836 |
|  |  | **T1w3D** | | | | **T1wSEM** | | | | **T2TSE** | | | |  |  |  |  |
| **CN:CA** | Age | 0.001 | 0.000 | 0.001 | **0.032*** | 0.000 | -0.002 | 0.001 | 0.630 | -0.004 | -0.007 | -0.002 | **0.003**** |  |  |  |  |
|  | Group comparison | 0.011 | 0.001 | 0.022 | **0.031*** | 0.040 | 0.013 | 0.067 | **0.004**** | 0.014 | -0.036 | 0.064 | 0.582 |  |  |  |  |
| **DN:PO** | Age | 0.000 | -0.001 | 0.001 | 0.926 | -0.002 | -0.003 | 0.000 | 0.070 | -0.001 | -0.003 | 0.001 | 0.170 |  |  |  |  |
|  | Group comparison | 0.015 | -0.002 | 0.031 | 0.080 | 0.019 | -0.010 | 0.048 | 0.191 | 0.030 | -0.003 | 0.063 | 0.073 |  |  |  |  |
| **NR:CA** | Age | -0.006 | -0.017 | 0.006 | 0.331 | -0.001 | -0.003 | 0.001 | 0.259 | -0.004 | -0.005 | -0002 | **0.000***** |  |  |  |  |
|  | Group comparison | -0.041 | -0.143 | 0.061 | 0.431 | -0.027 | -0.061 | 0.006 | 0.105 | -0.003 | -0.033 | 0.028 | 0.868 |  |  |  |  |
| **GP:CA** | Age | 0.000 | 0.000 | 0.001 | 0.480 | -0.001 | -0.002 | 0.001 | 0.255 | -0.007 | 0.000 | -0.005 | **0.000***** |  |  |  |  |
|  | Group comparison | 0.031 | 0.017 | 0.044 | **0.000***** | 0.036 | 0.011 | 0.061 | **0.005**** | -0.006 | -0.048 | 0.037 | 0.796 |  |  |  |  |
| **GP:TH** | Age | 0.000 | -0.001 | 0.001 | 0.807 | -0.001 | -0.002 | 0.000 | 0.163 | -0.005 | -0.007 | -0.003 | **0.000***** |  |  |  |  |
|  | Group comparison | 0.009 | -0.007 | 0.026 | 0.265 | 0.006 | -0.010 | 0.023 | 0.439 | -0.020 | -0.054 | 0.014 | 0.250 |  |  |  |  |
| **PU:CA** | Age | 0.000 | -0.002 | 0.001 | 0.751 | 0.000 | -0.002 | 0.001 | 0.725 | -0.005 | -0.007 | -0.002 | **0.001***** |  |  |  |  |
|  | Group comparison | 0.019 | -0.003 | 0.042 | 0.095 | 0.028 | 0.004 | 0.052 | **0.021*** | 0.009 | -0.036 | 0.054 | 0.679 |  |  |  |  |
| **SN:CA** | Age | 0.000 | -0.001 | 0.002 | **0.001***** | 0.000 | -0.001 | 0.002 | 0.425 | -0.005 | -0.006 | -0.003 | **0.000***** |  |  |  |  |
|  | Group comparison | -0.049 | -0.069 | -0.030 | **0.010**** | -0.030 | -0.069 | -0.030 | **0.000***** | -0.013 | -0.043 | 0.016 | 0.366 |  |  |  |  |
| **TH:CA** | Age | 0.000 | -0.001 | 0.001 | 0.712 | 0.000 | -0.002 | 0.001 | 0.848 | 0.000 | -0.002 | 0.001 | 0.570 |  |  |  |  |
|  | Group comparison | 0.022 | 0.005 | 0.038 | **0.010**** | 0.028 | 0.001 | 0.055 | **0.046*** | 0.023 | -0.006 | 0.052 | 0.119 |  |  |  |  |

| Table E2 | Multivariable analyses regarding the influence of the confounder "age" vs. the number of the GBCA dosages applied .a = no measurements. B = signal change per year. Sig. = significance. | | | | | | | | | | | | |  |  |  |  |
| --- | --- | --- | --- | --- | --- | --- | --- | --- | --- | --- | --- | --- | --- | --- | --- | --- | --- |
| **Sequence** |  | **B** | **95% Confidence Interval** | | **Sig.** | **B** | **95% Confidence Interval** | | **Sig.** | **B** | **95% Confidence Interval** | | **Sig.** | **B** | **95% Confidence Interval** | | **Sig.** |
|  |  |  | **Lower Bound** | **Upper Bound** |  |  | **Lower Bound** | **Upper Bound** |  |  | **Lower Bound** | **Upper Bound** |  |  | **Lower Bound** | **Upper Bound** |  |
|  |  | **Look-Locker T1 mapping** | | | | **Variable Flip-Angle T1 mapping** | | | | **T2 mapping** | | | | **R2* mapping** | | | |
| **CA** | Age | 1.011 | -0.040 | 2.062 | 0.059 | 0.869 | -0.421 | 2.159 | 0.184 | 0.105 | 0.008 | 0.202 | **0.033*** | -0.039 | -0.225 | 0.147 | 0.677 |
|  | Number | -2.097 | -4.051 | -0.143 | **0.036*** | 0.266 | -2.125 | 2.657 | 0.826 | -0.109 | -0.292 | 0.075 | 0.243 | 0.190 | -0.163 | 0.543 | 0.287 |
| **CN** | Age | -0.997 | -2.367 | 0.372 | 0.151 | 1.293 | -0.909 | 3.496 | 0.247 | -0.108 | -0.208 | -0.008 | **0.034*** | 0.109 | -0.100 | 0.318 | 0.302 |
|  | Number | -0.128 | -2.691 | 2.434 | 0.921 | 2.444 | -1.640 | 6.528 | 0.238 | -0.126 | -0.315 | 0.063 | 0.190 | 0.236 | -0.159 | 0.631 | 0.239 |
| **DN** | Age |  |  |  |  | -0.168 | -1.574 | 1.237 | 0.812 | -0.118 | -0.270 | 0.035 | 0.131 | 0.258 | 0.104 | 0.412 | **0.001**** |
|  | Number |  |  |  |  | -2.733 | -5.339 | -0.126 | **0.040*** | 0.037 | -0.252 | 0.327 | 0.799 | -0.114 | -0.406 | 0.178 | 0.440 |
| **GP** | Age | -1.102 | -2.281 | 0.077 | 0.067 | -0.139 | -1.766 | 1.488 | 0.866 | -0.123 | -0.265 | 0.019 | 0.089 | 0.292 | 0.150 | 0.435 | **0.000***** |
|  | Number | -2.548 | -4.755 | -0.340 | **0.024*** | -1.527 | -4.543 | 1.489 | 0.317 | -0.274 | -0.543 | -0.005 | **0.046*** | 0.104 | -0.165 | 0.373 | 0.445 |
| **NR** | Age | -0.349 | -1.711 | 1.012 | 0.610 | -0.431 | -1.723 | 0.861 | 0.509 | -0.131 | -0.268 | 0.007 | 0.063 | 0.184 | 0.077 | 0.291 | **0.001**** |
|  | Number | 0.662 | -1.860 | 3.185 | 0.601 | 1.307 | -1.089 | 3.703 | 0.281 | -0.233 | -0.494 | 0.028 | 0.080 | 0.101 | -0.102 | 0.303 | 0.327 |
| **PO** | Age |  |  |  |  | -1.283 | -2.917 | 0.351 | 0.122 | -0.131 | -0.197 | 0.188 | 0.967 | -0.088 | -0.343 | 0.166 | 0.492 |
|  | Number |  |  |  |  | -3.454 | -6.484 | -0.424 | **0.026*** | -0.233 | -0.453 | 0.276 | 0.631 | 0.474 | -0.008 | 0.956 | 0.054 |
| **PU** | Age | -0.728 | -1.895 | 0.440 | 0.219 | 0.759 | -1.676 | 3.195 | 0.537 | -0.004 | -0.217 | 0.049 | 0.211 | 0.068 | -0.043 | 0.226 | 0.178 |
|  | Number | -0.176 | -2.362 | 2.009 | 0.873 | 2.776 | -1.739 | 7.291 | 0.225 | -0.088 | -0.383 | 0.121 | 0.304 | 0.128 | -0.149 | 0.360 | 0.413 |
| **SN** | Age | -0.334 | -1.511 | 0.843 | 0.572 | -2.358 | -4.003 | -0.713 | **0.005**** | -0.084 | -0.289 | -0.006 | **0.042*** | 0.189 | 0.044 | 0.333 | **0.011**** |
|  | Number | -1.717 | -3.898 | 0.463 | 0.120 | -0.263 | -3.313 | 2.787 | 0.865 | -0.131 | -0.238 | 0.299 | 0.822 | -0.037 | -0.311 | 0.236 | 0.788 |
| **TH** | Age | 0.615 | -0.524 | 1.754 | 0.286 | 0.470 | -1.128 | 2.068 | 0.561 | -0.147 | 0.025 | 0.215 | **0.014*** | -0.003 | -0.036 | 0.031 | 0.864 |
|  | Number | 0.786 | -1.345 | 2.918 | 0.466 | -0.269 | -3.232 | 2.694 | 0.857 | 0.031 | -0.361 | -0.002 | **0.047*** | -0.011 | -0.075 | 0.052 | 0.720 |
|  |  | **Look-Locker T1 mapping** | | | | **Variable Flip-Angle T1 mapping** | | | | **T2 mapping** | | | | **R2* mapping** | | | |
| **CN:CA** | Age | -0.003 | -0.006 | -0.001 | **0.019*** | 0.000 | -0.003 | 0.003 | 0.838 | -0.003 | -0.005 | -0.001 | **0.003**** | 0.004 | -0.002 | 0.011 | 0.210 |
|  | Number | 0.003 | -0.002 | 0.008 | 0.265 | 0.002 | -0.004 | 0.007 | 0.475 | 0.000 | -0.004 | 0.004 | 0.847 | 0.003 | -0.009 | 0.015 | 0.659 |
| **DN:PO** | Age |  |  |  |  | 0.001 | -0.001 | 0.003 | 0.324 | -0.002 | -0.004 | 0.000 | 0.095 | 0.012 | 0.003 | 0.022 | **0.010**** |
|  | Number |  |  |  |  | 0.000 | -0.003 | 0.004 | 0.773 | 0.001 | -0.002 | 0.005 | 0.421 | -0.022 | -0.040 | -0.004 | **0.016*** |
| **NR:PO** | Age | -0.003 | -0.006 | -0.001 | **0.007**** | -0.002 | -0.004 | 0.000 | 0.124 | -0.003 | -0.006 | -0.001 | **0.004**** | 0.006 | -0.001 | 0.013 | 0.069 |
|  | Number | 0.005 | 0.000 | 0.009 | **0.036*** | 0.001 | -0.003 | 0.005 | 0.578 | -0.002 | -0.007 | 0.002 | 0.298 | 0.000 | -0.013 | 0.013 | 0.982 |
| **GP:CA** | Age | -0.003 | -0.005 | -0.001 | **0.012**** | -0.001 | -0.004 | 0.001 | 0.195 | -0.003 | -0.005 | -0.001 | **0.006**** | 0.009 | 0.003 | 0.016 | **0.007**** |
|  | Number | -0.001 | -0.005 | 0.003 | 0.744 | -0.002 | -0.006 | 0.002 | 0.318 | -0.003 | -0.007 | 0.001 | 0.163 | 0.000 | -0.012 | 0.013 | 0.989 |
| **GP:TH** | Age | -0.002 | -0.004 | 0.000 | 0.047 | -0.001 | -0.002 | 0.001 | 0.568 | -0.003 | -0.005 | -0.001 | **0.003**** | 0.013 | 0.007 | 0.019 | **0.000***** |
|  | Number | -0.004 | -0.007 | 0.000 | 0.043 | -0.001 | -0.005 | 0.002 | 0.495 | -0.002 | -0.006 | 0.002 | 0.361 | 0.006 | -0.006 | 0.017 | 0.325 |
| **PU:CA** | Age | -0.001 | -0.003 | 0.001 | 0.382 | -0.001 | -0.004 | 0.003 | 0.721 | -0.003 | -0.005 | 0.000 | **0.025*** | 0.003 | -0.003 | 0.009 | 0.348 |
|  | Number | 0.003 | -0.001 | 0.008 | 0.097 | 0.002 | -0.004 | 0.008 | 0.435 | -0.001 | -0.005 | 0.004 | 0.750 | 0.001 | -0.011 | 0.013 | 0.910 |
| **SN:CA** | Age | -0.003 | -0.005 | -0.001 | **0.003**** | -0.004 | -0.006 | -0.001 | **0.006**** | -0.003 | -0.006 | -0.001 | **0.005**** | 0.007 | -0.001 | 0.015 | 0.095 |
|  | Number | 0.001 | -0.003 | 0.005 | 0.505 | -0.001 | -0.005 | 0.004 | 0.795 | 0.001 | -0.003 | 0.006 | 0.573 | -0.003 | -0.018 | 0.012 | 0.675 |
| **TH:CA** | Age | -0.003 | -0.005 | 0.000 | 0.059 | -0.001 | -0.003 | 0.002 | 0.630 | 0.000 | -0.002 | 0.002 | 0.987 | 0.000 | -0.005 | 0.005 | 0.896 |
|  | Number | 0.003 | -0.002 | 0.008 | 0.181 | -0.001 | -0.005 | 0.004 | 0.745 | 0.000 | -0.005 | 0.002 | 0.478 | -0.001 | -0.011 | 0.008 | 0.757 |
|  |  | **T1w 3D (MPRAGE)** | | | | **T1w SEM** | | | | **T2w TSE** | | | |  |  |  |  |
| **CN:CA** | Age | 0.001 | 0.000 | 0.001 | **0.041*** | 0.000 | -0.002 | 0.001 | 0.541 | 0.000 | -0.002 | 0.001 | 0.541 |  |  |  |  |
|  | Number | 0.001 | 0.000 | 0.002 | 0.081 | 0.004 | 0.001 | 0.007 | **0.009**** | 0.004 | 0.001 | 0.007 | **0.009**** |  |  |  |  |
| **DN:PO** | Age | 0.000 | -0.001 | 0.001 | 0.780 | -0.002 | -0.003 | 0.000 | 0.062 | -0.002 | -0.003 | 8.529E-05 | 0.062 |  |  |  |  |
|  | Number | 0.002 | 0.000 | 0.004 | **0.040*** | 0.002 | -0.001 | 0.005 | 0.205 | 0.002 | -0.001 | 0.005 | 0.205 |  |  |  |  |
| **NR:PO** | Age | 0.000 | -0.001 | 0.001 | 0.674 | -0.001 | -0.003 | 0.001 | 0.256 | -0.001 | -0.003 | 0.001 | 0.256 |  |  |  |  |
|  | Number | 0.003 | 0.001 | 0.005 | **0.000***** | 0.002 | -0.001 | 0.006 | 0.215 | 0.002 | -0.001 | 0.006 | 0.215 |  |  |  |  |
| **GP:CA** | Age | 0.000 | -0.001 | 0.001 | 0.697 | -0.001 | -0.002 | 0.001 | 0.226 | -0.001 | -0.002 | 0.001 | 0.226 |  |  |  |  |
|  | Number | 0.003 | 0.002 | 0.005 | **0.000***** | 0.003 | 0.001 | 0.006 | **0.017*** | 0.003 | 0.001 | 0.006 | **0.017*** |  |  |  |  |
| **GP:TH** | Age | 0.000 | -0.001 | 0.001 | 0.965 | -0.001 | -0.002 | 0.000 | 0.163 | -0.005 | -0.007 | -0.003 | **0.000***** |  |  |  |  |
|  | Number | 0.002 | 0.000 | 0.003 | 0.094 | 0.001 | -0.001 | 0.002 | 0.530 | -0.002 | -0.006 | 0.001 | 0.223 |  |  |  |  |
| **PU:CA** | Age | 0.000 | -0.002 | 0.001 | 0.689 | 0.000 | -0.002 | 0.001 | 0.632 | 0.000 | -0.002 | 0.001 | 0.632 |  |  |  |  |
|  | Number | 0.002 | -0.001 | 0.005 | 0.123 | 0.003 | 0.000 | 0.006 | **0.028*** | 0.003 | 0.000 | 0.006 | **0.028*** |  |  |  |  |
| **SN:CA** | Age | 0.000 | -0.001 | 0.001 | 0.606 | -0.001 | -0.003 | 0.001 | 0.343 | -0.001 | -0.003 | 0.001 | 0.343 |  |  |  |  |
|  | Number | 0.005 | 0.003 | 0.007 | **0.000***** | 0.003 | 0.000 | 0.006 | 0.076 | 0.003 | 0.000 | 0.006 | 0.076 |  |  |  |  |
| **TH:CA** | Age | 0.000 | -0.001 | 0.001 | 0.745 | 0.000 | -0.002 | 0.001 | 0.794 | 0.000 | -0.002 | 0.001 | 0.794 |  |  |  |  |
|  | Number | 0.002 | 0.000 | 0.004 | 0.056 | 0.003 | 0.000 | 0.006 | 0.088 | 0.003 | 0.000 | 0.006 | 0.088 |  |  |  |  |

| Table E3 | Multivariable analyses regarding the influence of the confounder "age" vs. the number of the GBCA dosages applied .a = no measurements. B = signal change per year. Sig. = significance. | | | | | | | | | | | | |  |  |  |  |
| --- | --- | --- | --- | --- | --- | --- | --- | --- | --- | --- | --- | --- | --- | --- | --- | --- | --- |
| **Sequence** |  | **B** | **95% Confidence Interval** | | **Sig.** | **B** | **95% Confidence Interval** | | **Sig.** | **B** | **95% Confidence Interval** | | **Sig.** | **B** | **95% Confidence Interval** | | **Sig.** |
|  |  |  | **Lower Bound** | **Upper Bound** |  |  | **Lower Bound** | **Upper Bound** |  |  | **Lower Bound** | **Upper Bound** |  |  | **Lower Bound** | **Upper Bound** |  |
|  |  | **Look-Locker T1 mapping** | | | | **Variable Flip-Angle T1 mapping** | | | | **T2 mapping** | | | | **R2* mapping** | | | |
| **CA** | Age | 1.004 | -0.054 | 2.063 | 0.063 | 0.873 | -0.418 | 2.165 | 0.183 | 0.105 | 0.008 | 0.202 | **0.035*** | -0.036 | -0.223 | 0.151 | 0.705 |
|  | Volume | -0.137 | -0.276 | 0.002 | 0.054 | 0.015 | -0.155 | 0.186 | 0.858 | -0.007 | -0.020 | 0.006 | 0.284 | 0.011 | -0.014 | 0.036 | 0.385 |
| **CN** | Age | -0.986 | -2.358 | 0.386 | 0.157 | 1.321 | -0.890 | 3.532 | 0.238 | -0.107 | -0.207 | -0.007 | **0.036*** | 0.106 | -0.103 | 0.314 | 0.318 |
|  | Volume | -0.016 | -0.198 | 0.167 | 0.865 | 0.150 | -0.142 | 0.442 | 0.309 | -0.009 | -0.023 | 0.004 | 0.171 | 0.018 | -0.010 | 0.047 | 0.197 |
| **DN** | Age |  |  |  |  | -0.172 | -1.583 | 1.240 | 0.810 | -0.115 | -0.268 | 0.038 | 0.140 | 0.258 | 0.104 | 0.412 | **0.001***** |
|  | Volume |  |  |  |  | -0.185 | -0.371 | 0.001 | 0.051 | 0.001 | -0.020 | 0.022 | 0.918 | -0.008 | -0.029 | 0.013 | 0.438 |
| **GP** | Age | -1.089 | -2.270 | 0.093 | 0.070 | -0.153 | -1.784 | 1.479 | 0.853 | -0.124 | -0.266 | 0.019 | 0.088 | 0.290 | 0.148 | 0.432 | **0.000***** |
|  | Volume | -0.182 | -0.339 | -0.025 | **0.024*** | -0.096 | -0.311 | 0.119 | 0.379 | -0.018 | -0.038 | 0.001 | 0.061 | 0.009 | -0.011 | 0.028 | 0.377 |
| **NR** | Age | -0.346 | -1.705 | 1.014 | 0.612 | -0.458 | -1.750 | 0.833 | 0.483 | -0.129 | -0.266 | 0.009 | 0.067 | 0.185 | 0.078 | 0.292 | **0.001***** |
|  | Volume | 0.047 | -0.132 | 0.226 | 0.603 | 0.107 | -0.064 | 0.277 | 0.216 | -0.017 | -0.036 | 0.001 | 0.070 | 0.006 | -0.008 | 0.021 | 0.392 |
| **PO** | Age |  |  |  |  | -1.269 | -2.906 | 0.368 | 0.127 | -0.004 | -0.197 | 0.189 | 0.965 | -0.087 | -0.342 | 0.169 | 0.502 |
|  | Volume |  |  |  |  | -0.246 | -0.462 | -0.030 | **0.026*** | -0.006 | -0.032 | 0.020 | 0.651 | 0.032 | -0.003 | 0.066 | 0.071 |
| **PU** | Age | -0.755 | -1.925 | 0.414 | 0.203 | 0.674 | -1.755 | 3.103 | 0.583 | -0.089 | -0.223 | 0.044 | 0.188 | 0.093 | -0.042 | 0.227 | 0.176 |
|  | Volume | 0.005 | -0.151 | 0.161 | 0.952 | 0.245 | -0.076 | 0.565 | 0.133 | -0.006 | -0.024 | 0.012 | 0.500 | 0.007 | -0.011 | 0.025 | 0.458 |
| **SN** | Age | -0.369 | -1.552 | 0.814 | 0.535 | -2.413 | -4.061 | -0.765 | **0.005**** | -0.145 | -0.287 | -0.003 | **0.046*** | 0.192 | 0.048 | 0.337 | **0.010**** |
|  | Volume | -0.101 | -0.257 | 0.054 | 0.198 | 0.017 | -0.201 | 0.234 | 0.878 | 0.001 | -0.018 | 0.020 | 0.943 | -0.005 | -0.024 | 0.015 | 0.633 |
| **TH** | Age | 0.639 | -0.504 | 1.782 | 0.270 | 0.512 | -1.087 | 2.112 | 0.526 | 0.119 | 0.024 | 0.214 | **0.014*** | -0.004 | -0.038 | 0.030 | 0.816 |
|  | Volume | 0.039 | -0.113 | 0.191 | 0.611 | -0.046 | -0.256 | 0.165 | 0.669 | -0.012 | -0.025 | 0.001 | 0.061 | 0.000 | -0.005 | 0.004 | 0.941 |
|  |  | **Look-Locker T1 mapping** | | | | **Variable Flip-Angle T1 mapping** | | | | **T2 mapping** | | | | **R2* mapping** | | | |
| **CN:CA** | Age | -0.003 | -0.006 | 0.000 | **0.020*** | 0.000 | -0.003 | 0.003 | 0.848 | -0.003 | -0.005 | -0.001 | **0.004**** | 0.884 | 0.614 | 1.154 | **0.000***** |
|  | Volume | 0.000 | 0.000 | 0.001 | 0.341 | 0.000 | 0.000 | 0.001 | 0.532 | 0.000 | 0.000 | 0.000 | 0.773 | 0.004 | -0.003 | 0.010 | 0.233 |
| **DN:PO** | Age |  |  |  |  | 0.001 | -0.001 | 0.003 | 0.332 | -0.002 | -0.004 | 0.000 | 0.104 | 0.012 | 0.003 | 0.022 | **0.012*** |
|  | Volume |  |  |  |  | 0.000 | 0.000 | 0.000 | 0.728 | 0.000 | 0.000 | 0.000 | 0.552 | -0.001 | -0.003 | 0.000 | **0.029*** |
| **NR:PO** | Age | -0.003 | -0.006 | -0.001 | **0.008**** | -0.002 | -0.004 | 0.000 | 0.116 | -0.003 | -0.006 | -0.001 | **0.005**** | 0.006 | -0.001 | 0.013 | 0.073 |
|  | Volume | 0.000 | 0.000 | 0.001 | **0.050*** | 0.000 | 0.000 | 0.000 | 0.479 | 0.000 | 0.000 | 0.000 | 0.259 | 0.000 | -0.001 | 0.001 | 0.909 |
| **GP:CA** | Age | -0.003 | -0.005 | -0.001 | **0.013*** | -0.001 | -0.004 | 0.001 | 0.190 | -0.003 | -0.006 | -0.001 | **0.006**** | 0.009 | 0.002 | 0.016 | **0.008**** |
|  | Volume | 0.000 | 0.000 | 0.000 | 0.665 | 0.000 | 0.000 | 0.000 | 0.384 | 0.000 | -0.001 | 0.000 | 0.185 | 0.000 | -0.001 | 0.001 | 0.797 |
| **GP:TH** | Age | -0.002 | -0.004 | 0.000 | **0.047*** | -0.001 | -0.003 | 0.001 | 0.536 | -0.003 | -0.005 | -0.001 | **0.003**** | 0.013 | 0.007 | 0.019 | **0.000***** |
|  | Volume | 0.000 | -0.001 | 0.000 | 0.058 | 0.000 | 0.000 | 0.000 | 0.680 | 0.000 | 0.000 | 0.000 | 0.394 | 0.000 | 0.000 | 0.001 | 0.320 |
| **PU:CA** | Age | -0.003 | -0.005 | 0.000 | 0.057 | -0.001 | -0.004 | 0.003 | 0.677 | -0.003 | -0.005 | 0.000 | **0.022*** | 0.003 | -0.003 | 0.009 | 0.357 |
|  | Volume | 0.000 | 0.000 | 0.001 | 0.179 | 0.000 | 0.000 | 0.001 | 0.298 | 0.000 | 0.000 | 0.000 | 0.940 | 0.000 | -0.001 | 0.001 | 0.853 |
| **SN:CA** | Age | -0.003 | -0.005 | -0.001 | **0.003**** | -0.004 | -0.006 | -0.001 | **0.005**** | -0.003 | -0.006 | -0.001 | **0.006**** | 0.909 | 0.573 | 1.244 | **0.000***** |
|  | Volume | 0.000 | 0.000 | 0.000 | 0.474 | 0.000 | 0.000 | 0.000 | 0.993 | 0.000 | 0.000 | 0.000 | 0.702 | 0.007 | -0.001 | 0.015 | 0.095 |
| **TH:CA** | Age | -0.001 | -0.003 | 0.001 | 0.401 | -0.001 | -0.003 | 0.002 | 0.652 | 0.000 | -0.002 | 0.002 | 0.985 | 0.000 | -0.005 | 0.005 | 0.930 |
|  | Volume | 0.000 | 0.000 | 0.000 | 0.156 | 0.000 | 0.000 | 0.000 | 0.645 | 0.000 | 0.000 | 0.000 | 0.486 | 0.000 | -0.001 | 0.001 | 0.916 |
|  |  | **T1w 3D (MPRAGE)** | | | | **T1w SEM** | | | | **T2TSE** | | | |  |  |  |  |
| **CN:CA** | Age | 0.001 | 0.000 | 0.001 | **0.048*** | -0.001 | -0.002 | 0.001 | 0.510 | -0.004 | -0.007 | -0.002 | **0.004**** |  |  |  |  |
|  | Volume | 0.000 | 0.000 | 0.000 | **0.043*** | 0.000 | 0.000 | 0.001 | **0.006**** | 0.00008 | 0.000 | 0.000 | 0.691 |  |  |  |  |
| **DN:PO** | Age | 0.000 | -0.001 | 0.001 | 0.793 | -0.002 | -0.003 | 0.000 | 0.066 | -0.001 | -0.003 | 0.000 | 0.131 |  |  |  |  |
|  | Volume | 0.000 | 0.000 | 0.000 | 0.055 | 0.000 | 0.000 | 0.000 | 0.255 | 0.000 | -0.000 | 0.001 | 0.060 |  |  |  |  |
| **NR:PO** | Age | 0.000 | -0.001 | 0.001 | 0.748 | -0.001 | -0.003 | 0.001 | 0.232 | -0.004 | -0.005 | -0.002 | **0.000***** |  |  |  |  |
|  | Volume | 0.000 | 0.000 | 0.000 | **0.000***** | 0.000 | 0.000 | 0.000 | 0.144 | 0.00002 | 0.000 | 0.000 | 0.894 |  |  |  |  |
| **GP:CA** | Age | 0.000 | -0.001 | 0.001 | 0.745 | -0.001 | -0.002 | 0.001 | 0.196 | -0.007 | -0.010 | -0.005 | **0.000***** |  |  |  |  |
|  | Volume | 0.000 | 0.000 | 0.000 | **0.000***** | 0.000 | 0.000 | 0.000 | **0.008**** | -0.00003 | 0.000 | 0.000 | 0.873 |  |  |  |  |
| **GP:TH** | Age | 0.000 | -0.001 | 0.001 | 0.926 | -0.001 | -0.002 | 0.000 | 0.154 | -0.005 | -0.007 | -0.003 | **0.000***** |  |  |  |  |
|  | Volume | 0.000 | 0.000 | 0.000 | 0.161 | 0.000 | 0.000 | 0.000 | 0.453 | 0.000 | 0.000 | 0.000 | 0.211 |  |  |  |  |
| **PU:CA** | Age | 0.000 | -0.002 | 0.001 | 0.723 | 0.000 | -0.002 | 0.001 | 0.599 | -0.005 | -0.007 | -0.002 | **0.001***** |  |  |  |  |
|  | Volume | 0.000 | 0.000 | 0.000 | 0.194 | 0.000 | 0.000 | 0.000 | **0.020*** | 0.000 | 0.000 | 0.000 | 0.550 |  |  |  |  |
| **SN:CA** | Age | 0.000 | -0.001 | 0.001 | 0.623 | -0.001 | -0.003 | 0.001 | 0.306 | -0.005 | -0.006 | -0.003 | **0.000***** |  |  |  |  |
|  | Volume | 0.000 | 0.000 | 0.001 | **0.000***** | 0.000 | 0.000 | 0.000 | **0.041*** | 0.000 | 0.000 | 0.000 | 0.185 |  |  |  |  |
| **TH:CA** | Age | 0.000 | -0.001 | 0.001 | 0.830 | 0.000 | -0.002 | 0.001 | 0.756 | -0.001 | -0.002 | 0.001 | 0.457 |  |  |  |  |
|  | Volume | 0.000 | 0.000 | 0.000 | **0.018** | 0.000 | 0.000 | 0.000 | 0.062 | 0.000 | 0.000 | 0.000 | 0.065 |  |  |  |  |
